# Evaluation of a multi-species Protein A-ELISA assay for plague serologic diagnosis in humans and other mammal hosts

**DOI:** 10.1371/journal.pntd.0009805

**Published:** 2022-05-12

**Authors:** Matheus Filgueira Bezerra, Camila Cavalcanti Xavier, Alzira Maria Paiva de Almeida, Christian Robson de Souza Reis

**Affiliations:** Departamento de Microbiologia – Instituto Aggeu Magalhães; FIOCRUZ -PE. Recife, Brazil; Institut Pasteur, FRANCE

## Abstract

**Background:**

The Hemagglutination assay (HA) is widely used in plague diagnosis, however, it has a subjective interpretation and demands high amounts of antigen and other immunobiological supplies. On the other hand, the conventional Anti-IgG ELISA is limited by the need of specific conjugates for multiple plague hosts, which leaves a gap for new diagnostic methods able to cover both the diagnosis of human cases and the epidemiological surveillance of multiple sentinel species.

**Methods:**

We developed an ELISA Protein A-peroxidase method to detect anti-F1 antibodies across several species, including humans. To determine the cut-off and performance rates, HA results from 288 samples (81 rabbits, 64 humans, 66 rodents and 77 dogs) were used as reference. Next, we evaluated the agreement between Protein A-ELISA and Anti-IgG ELISA in an expanded sample set (n = 487).

**Results:**

Optimal conditions were found with 250ng/well of F1 and 1:500 serum dilution. Protein A-ELISA showed high repeatability and reproducibility. We observed good correlation rates between the Protein A and IgG ELISAs optical densities and a higher positive/negative OD ratio for the Protein A-ELISA method. The overall sensitivity, specificity and area under the curve for Protein A-ELISA were 94%, 99% and 0.99, respectively. Similar results were observed for each species separately. In the analysis of the expanded sample set, there was a strong agreement between Protein A and IgG assays (kappa = 0.97). Furthermore, there was no cross-reaction with other common infectious diseases, such as dengue, Zika, Chagas disease, tuberculosis (humans) and ehrlichiosis, anaplasmosis and leishmaniasis (dogs).

**Conclusions:**

Altogether, the Protein A-ELISA showed high performance when compared both to HA and Anti-IgG ELISA, with a polyvalent single protocol that requires reduced amounts of antigen and can be employed to any plague hosts.

## Author summary

Here, we developed and evaluated an ELISA diagnostic test based on the Protein A-peroxidase conjugate that allows the test to be used for plague laboratorial diagnosis not only in humans, but also in a wide range of mammalian species. This particularity is specifically important for plague epidemiological surveillance, given that *Yersinia pestis*, the causative agent of plague, have a long list of animal reservoirs across distinct ecosystems. Briefly, we first evaluated the best reaction parameters, such as antigen concentration, serum and protein A-conjugate dilutions. Next, we used serum samples from humans, dogs, rodents and rabbits (n = 288) with known results for plague serology by a conventional method, to evaluate the performance of the new Protein A-ELISA test. We observed a good performance of the novel Protein A-ELISA test, with high sensitivity and specificity rates. Evaluation of the coefficient of variation revealed that the test measurements suffer little variation, and therefore, has high repeatability and reproducibility. Next, by evaluating 487 samples, we observed a high degree of concordance between the Protein A-ELISA with a conventional IgG-based ELISA. Furthermore, this test showed no significant cross-reaction with other common infectious diseases. Altogether, the Protein A-ELISA showed high performance when compared both to HA and Anti-IgG ELISA, with a single protocol that requires reduced amounts of antigen and can be employed to several plague hosts.

## Introduction

Plague is a flea-transmitted disease caused by the gram-negative bacterium *Yersinia pestis* and was responsible for at least three pandemics in the past [1]. Although nowadays plague can be treated with antibiotics, there is a lack of vaccines able to provide long-term immunity and this disease still threaten individuals living in remote places, close to wildlife hosts but distant from specialized healthcare services [2]. Human cases and deaths are recorded annually in several countries across Africa, Asia and the Americas [3]. Despite the declining incidence worldwide, the interest in plague is constant because of its potential to establish new epidemics and application as a biological weapon [2,4].

Although the rodents are the main plague reservoir, practically any mammal can be infected by *Y*. *pestis* and may take part in the dynamics of the infection [5]. An interesting feature of plague is that, under certain conditions, the disease is able to remain quiescent in the natural foci for decades and eventually reemerge among the wild fauna and spillover to human populations [6,7,8]. Due to this unique feature, it is of utmost importance to perform continuous monitoring of plague areas. In this regard, serological methods are an important surveillance tool, as it identifies not only animals with the active form of the disease, but also those previously exposed [9]. Most serological tests for plague are based on the detection of antibodies against the F1 capsular antigen, which is exclusive to *Y*. *pestis*, and highly immunogenic for humans and other mammals [10,11].

Given its polyvalence for sera from all taxonomic family groups, hemagglutination (HA) has been widely used for plague serological diagnosis for several decades [10,11,12]. However, some commonly observed problems in HA, such as interpretations bias, cross-reaction with other infections, high consumption of F1 antigen and use of perishable biological supplies, led many laboratories to migrate to IgG ELISA tests [13–17]. On the other hand, conventional ELISA requires a specific anti-IgG conjugate and different optimization for each mammal species. Thus, there is a need of new diagnostic methods that can improve the diagnosis and epidemiological surveillance of human and animal plague across the globe [2,4,18].

Alternatively to immunoglobulin (anti-IgG) conjugates, the *Staphylococcus aureus* protein A has been proposed for diagnosis of other multi-host diseases due to its universal affinity for immunoglobulins from various species of domestic and wild mammals [19–21]. To tackle this gap, we proposed a Protein A-based indirect ELISA method, able to detect anti-F1 antibodies from humans and other plague hosts within a single protocol.

## Methods

### Ethics statement

The production of immune sera for positive controls was approved by the Animal Ethics Committee of the Aggeu Magalhães Institute (CEUA/Fiocruz, protocol number: L-020/09), following the local Animal Ethics Committees (CEUA/IAM) guidelines and it supplies the laboratorial diagnosis of plague by the SRP. The use of the retrospectively collected human serum samples in this research was approved by Ethics Committee of the Aggeu Magalhães Institute (CEP/IAM/FIOCRUZ-PE, protocol number: CAAE 50163615.8.0000.5190). Sera from rodents and dogs were obtained during the routine operations of the Brazilian plague surveillance program and are maintained in the serum collection of the SRP-IAM.

### F1 production

**Fig 1:**
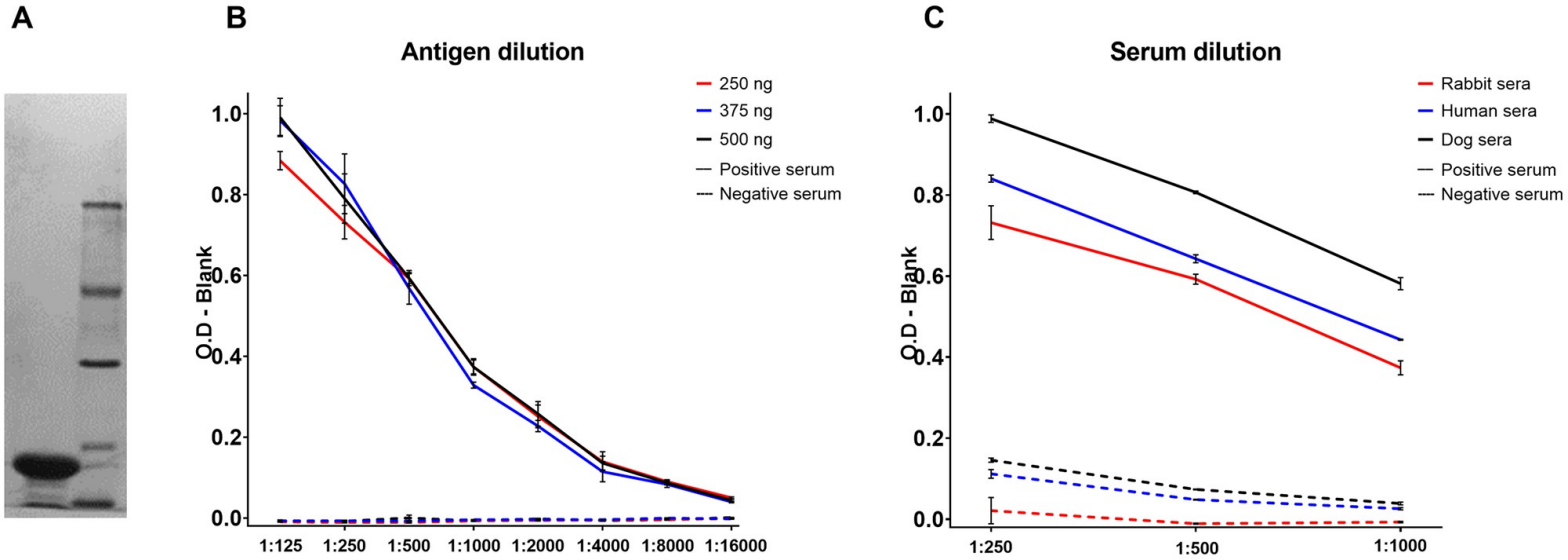
Standardization of Protein A-ELISA. F1 antigen purified from the A1122 *Y*. *pestis* strain in culture **(A)**. Three concentrations of F1 antigen (250 ng, 375 ng and 500 ng) were tested. The assay was optimized at the concentration of 250 ng of F1 antigen per well **(B)**. Graph with the optical densities from titrated sera in different species **(C)**.

The F1 antigen was extracted from the attenuated A1122 *Y*. *pestis* strain (pCD1-), according to the protocol described by Chu [10], in a Biosafety level 3 facility. The purified product was then mixed with 2× Laemmli buffer (1:1) containing 5% β-mercaptoethanol, heated at 100 °C for five minutes and loaded onto a 20% polyacrylamide gel (Fig 1A). The F1 antigen was quantified using the NanoDrop One Microvolume UV-Vis Spectrophotometer (Thermo-Fischer, USA).

### Sera samples

Initially, we retrospectively accessed 288 sera (98 positives and 190 negatives) including 81 control rabbits (37 positives and 44 negatives), 64 humans (21 positives and 43 negatives), 66 rodents (20 positives and 46 negatives) and 77 dogs (20 positives and 57 negatives) with well-characterized results in HA for cut-off determination and Protein A-ELISA validation. Next, 265 additional samples with unknown HA results nor used in cut-off determination were included to evaluate the agreement between the Protein A and IgG methods in ELISA. Sera were provided by the Brazilian Plague Reference Service (SRP) from the Aggeu Magalhães Institute (IAM) and originated mainly from the routine surveillance of the Brazilian plague areas including human cases, several rodent species and domestic carnivores (stray dogs) that prey on rodents [22,23]. Moreover, to evaluate cross-reaction with other common pathogens, we evaluated additional human and dog samples from individuals with other confirmed infections, such as dengue, Zika, Chagas disease, tuberculosis (humans) and ehrlichiosis, anaplasmosis and leishmaniasis (dogs).

The set of 21 HA-positive samples originated from human cases that occurred in the Brazilian states of Ceará, Pernambuco, Paraiba, Bahia and Minas Gerais between 1978 and 1995. These patients inhabited rural areas and underwent initial clinical and epidemiological investigation with suspected diagnosis of plague and had their diagnosis confirmed by either positive serology (HA, S4 Fig) alone or combination of positive serology and bacteriology (culture + bacteriophage lysis test). Out of 21 patients, we were able to retrieve the reported dates from disease onset for 13 cases. For those cases, the time interval between symptoms onset and serum collection ranged from 2 to 57 days, (median = 18 days, S2 Table). The negative human group constituted sera from suspected cases that tested negative in the routine laboratory diagnosis of plague [24,25].

The rodents’ samples comprised the species *Calomys callosus* (n = 18), *Cerradomys langguthi* (n = 1), *Mus musculus* (n = 20), *Necromys lasiurus* (n = 10), *Thrichomys laurentius* (n = 4) and *Rattus rattus* (n = 13). From these, 30 samples were animals captured in plague foci areas in the Brazilian state of Pernambuco during surveillance routine in 2019 and 2020 (all tested negative in both HA-HI and ELISA). The collection of the rodents was performed as described [26]. The other 36 samples were obtained from experimental subjects, including 20 *M*. *musculus* Swiss Webster mice (18 positives and 2 negatives) and 16 *C*. *callosus* (2 positives and 14 negatives) from colonies raised in the IAM facilities and submitted to Y. pestis inoculation for control on development and evaluation of diagnosis tests.

Sera from rabbits immunized with formol-killed *Y*. *pestis* and other pathogenic *Yersinia* strains (whole-cells immunization) or with the purified F1 antigen, produced as previously described [27] for positive control in routine diagnosis were kindly provided by the SRP. From the 37 positive control sera, 14 were from rabbits exposed to the reference EV76 or A1122 *Y*. *pestis* strains in independent experiments, 18 were from rabbits exposed to diverse Brazilian *Y*. *pestis* strains from the Fiocruz-CYP (http://cyp.fiocruz.br) bacterial cultures collection and five were from rabbits exposed to the purified F1 antigen (three native F1 and two recombinant F1, expressed in *E*. *coli*) [27]. Additionally, five rabbits immunized with distinct isolates of *Yersinia pseudotuberculosis* and two with *Yersinia enterocolitica* were included. The other 37 negative control rabbit sera were obtained from animals from the IAM facilities that did not underwent any experimental intervention.

### Protein A-ELISA and Anti-IgG ELISA

The ELISA tests were adapted from previously established protocols [10,14]. Briefly, 96-well plates (Techno Plastic Products, Switzerland) were incubated overnight with 250 ng of F1 diluted in 100 μl of a 0,05 M, pH 9,6 carbonate-bicarbonate buffer per well. Next, the plates were washed twice with 500 μl of PBS per well (PW 40 Microplate Washer, Bio-Rad, USA) and blocked with 100 μl of a 10% solution of low-fat milk in PBS for one hour. After a double wash with 500 μl of PBS-T (Tween 20, 0.05%), 100 μl of serum samples diluted (1:500) in a 10% milk/PBS-T solution were incubated in the plate at room temperature for one hour and washed twice with 500 μl of PBS-T. A 100 μl of Protein A–Peroxidase from *Staphylococcus aureus*/horseradish (Sigma-Aldrich, USA) or goat anti-human, rabbit or dog-peroxidase (Kirkegaard & Perry Laboratories, USA) diluted in 10% milk/PBS-T solution (1:10.000 and 1:2.500, respectively) were added and incubated at room temperature for one hour and washed twice with 500 μl of PBS-T. Finally, 150 μl of 2 mg/mL OPD (o-phenylenediamine dihydrochloride; Sigma–Aldrich) and 1:10^3^ H_2_O_2_ diluted in citrate-phosphate buffer (pH = 5.0) was incubated in each well for 30 minutes at room temperature in a dark environment. The reaction was stopped by the addition of 100 μl 2.5 M sulfuric acid (H_2_SO_4_) per well and plates were read at the optical density of 490nm (iMark Microplate Absorbance Reader, Bio-Rad, USA). In the ELISA Protein A and IgG standardization experiments, previously characterized positive sera with intermediate titers in HA were selected as a representative sample for each species.

All samples were measured in triplicates and the background (blank) optical density (OD) from each plate was subtracted from the average sample OD. Distinct concentrations of F1 antigen, peroxidase conjugates and sera dilution were tested to determine optimal conditions. The cut-offs were determined according to the best specificity/sensitivity (Youden’s index) from the Receiver Operating Characteristic (ROC) curve. A distinct cut-off was calculated for each peroxidase conjugate. Since the rodent samples included a rather heterogeneous range of wild species [22], we could not test them for Anti-IgG ELISA.

### Hemagglutination assay (HA)

The hemagglutination (HA) assay was performed as described previously [10]. In short, the F1 antigen was immobilized onto sheep red blood cells (SRBC) previously fixed with glutaraldehyde and tannic acid. Next, the F1-coated SRBC (25 μL/well) were incubated with the test serum serially diluted in eight wells starting from 1/4 in HA (0.85% saline + normal rabbit serum) buffer. The specificity of HA was accessed by the hemagglutination inhibition (HI). The test is considered positive when the HA endpoint is depressed by three or more HI dilutions (titers ≥ 1/16 are considered positive).

### Statistical analysis

The HA test, which is routinely used in the SRP, was used as the gold standard to calculate Protein-A ELISA and IgG ELISA performance rates. Sensitivity, specificity, accuracy and confidence intervals [28,29] were calculated using the https://www.medcalc.org platform. Receiver Operating Characteristic (ROC) curves, area under the curve (AUC), scatterplots and correlations were calculated to measure the ELISA test performance [29,30] using the GraphPad Prism version 5 software. Pearson test was used to measure the correlation between ODs from distinct tests and Mann-Whitney test was used to compare OD means. The intra and inter-assay variability was measured using the coefficient of variation (CV) from one serum from a rabbit immunized with the A1122 *Y*. *pestis* strain and one negative rabbit serum. Samples were tested in eight replicates within runs and across six experiments in non-consecutive days.

The Kappa test was initially applied to determine the agreement rate between the ELISA and HA tests (n = 288) and next, in an expanded sample set to measure the agreement rate between Protein A-ELISA and Anti-IgG ELISA (n = 487). The index was calculated using the Quickcalc GraphPad tool (https://www.graphpad.com/quickcalcs/kappa2). Statistical tests were applied with a 95% confidence interval.

## Results

### Standardization of Protein A-ELISA and Anti-IgG ELISA

The optimal conditions were determined for Protein A-ELISA by evaluating separately distinct amount of F1 antigen per well and serum dilutions. There was no significant difference between the ODs by using the amounts of 250, 375 and 500 ng per well (Fig 1B). Thus, we decided to establish the lowest amount (250 ng per well) for the subsequent experiments. By testing three serum dilutions (1: 250; 1: 500 and 1: 1000), the 1:500 dilution showed high ODs for positive samples and low background for negative samples (Fig 1C). For optimization of the three Anti-IgG ELISA tests (rabbit, human and dog), we maintained the amount of F1 antigen (250 ng/well) and sera dilution (1:500) previously established for Protein A-ELISA and tested four dilutions for IgG conjugate (1:1250; 1:2500; 1:5000 and 1:10000). We found the best positive/negative ratios at the 1:2.500 dilutions for all IgG conjugates (S1 Fig).

### Comparing ODs, cut-offs and cross-reaction between Protein A and IgG ELISAs

**Fig 2:**
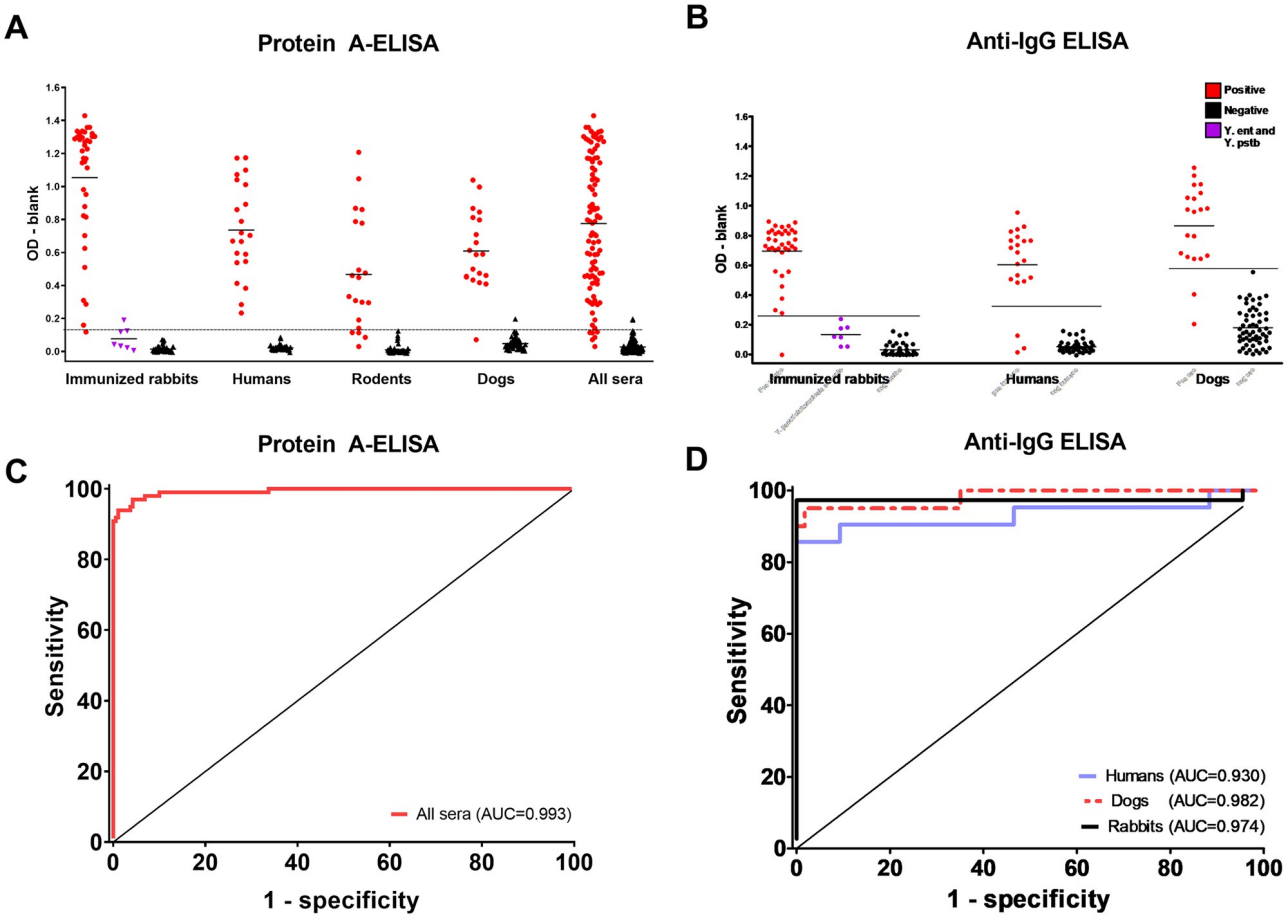
Validation of the diagnosis of plague by Protein A-ELISA and Anti-IgG ELISA in HA-tested sera. Comparison between Protein A-ELISA and IgG ODs from positive and negative sera previously tested for hemagglutination: the cut-offs values of Protein A-ELISA **(A**) and Anti-IgG ELISA **(B)** tests were determined at their best performance rates, where the ROC curves **(C-D)** reached its best sensitivity + specificity combination. The area under the curve values were close to 1.0, indicating a high capability of the test to distinguish negative and positive samples. ELISA cut-off values can be found at Table 1.

Whilst a single cut-off was established for Protein A-ELISA considering the best Youden’s index possible across all tested species, individual cut-offs were established for IgG anti-rabbit, anti-human and anti-dog ELISAs (Table 1 and Fig 2A and 2B). We observed low background signals in negative samples for Protein A, anti-rabbit and anti-human IgG conjugates, but a rather marked background in anti-dog IgG conjugate, resulting in a narrower window of opportunity for cut-off.

**Table 1:** Cut-offs and diagnostic performance of Protein A-ELISA and Anti-IgG ELISA.

	N (positives/negatives)	Cut-off	OD ratio* (positives/negatives)	Sensitivity (CI95%)	Specificity (CI95%)	Accuracy (CI95%)	Kappa index (CI95%)
Hemagglutination	288 (98/190)	≥1:16	-	ref	ref	ref	ref
Protein A-ELISA							
Rabbit	81 (37/44)	0.130	81.1	97.3 (85.8–99.9)	97.7 (88.0–99.9)	97.5 (91.4–99.7)	0.950 (0.882–1.000)
Human	64 (21/43)	0.130	34.2	100 (83.9–100)	100 (91.8–100)	100 (94.4–100)	1.000 (1.000–1.000)
Rodent**	66 (20/46)	0.130	51.7	80 (56.3–94.3)	100 (92.3–100)	93.9 (85.2–98.3)	0.848 (0.705–0.991)
Dog	77 (20/57)	0.130	12.8	95.0 (75.1–99.8)	98.2 (90.6–99.9)	97.4 (90.9–99.7)	0.932 (0.840–1.000)
All	288 (98/190)	0.130	28.7	93.9 (87.1–97.7)	98.9 (96.2–99.9)	97.2 (94.6–98.8)	0.938 (0.895–0.980)
Anti-IgG ELISA							
Rabbit	81 (37/44)	0.258	22.4	97.3 (85.8–99.9)	100 (92.0–100)	98.7 (93.3–99.9)	0.975 (0.927–1.000)
Human	64 (21/43)	0.320	11.8	85.7 (63.7–96.9)	100 (89.8–100)	95.3 (86.9–99.0)	0.851 (0.711–0.991)
Dog	77 (20/57)	0.573	4.85	90.0 (68.3–98.7)	100 (93.7–100)	97.4 (90.9–99.6)	0.930 (0.835–1.000)

* Ratio between average OD values from positive and negative sera, according to HA test. Samples were classified as positive or negative according to HA.

** Due to the wide range of species, rodents were tested only with the polyvalent tests Protein A-ELISA and HA.

Overall, the average ODs from positive samples were significantly higher than the ODs from negative samples both in protein A and in IgG tests (Mann-Whitney test p < 10^−3^). The ratios between the OD means from positive and negative samples were considerably higher for the protein A conjugate (all samples = 28.7; rabbit = 81.1; human = 34.2; rodent = 51.7 and dog = 12.8), when compared to anti-rabbit (22.4), anti-human (11.8) and anti-dog (4.9) IgG conjugates. OD ratios and averages are shown in Table 1 and S1 Table, respectively.

**Fig 3:**
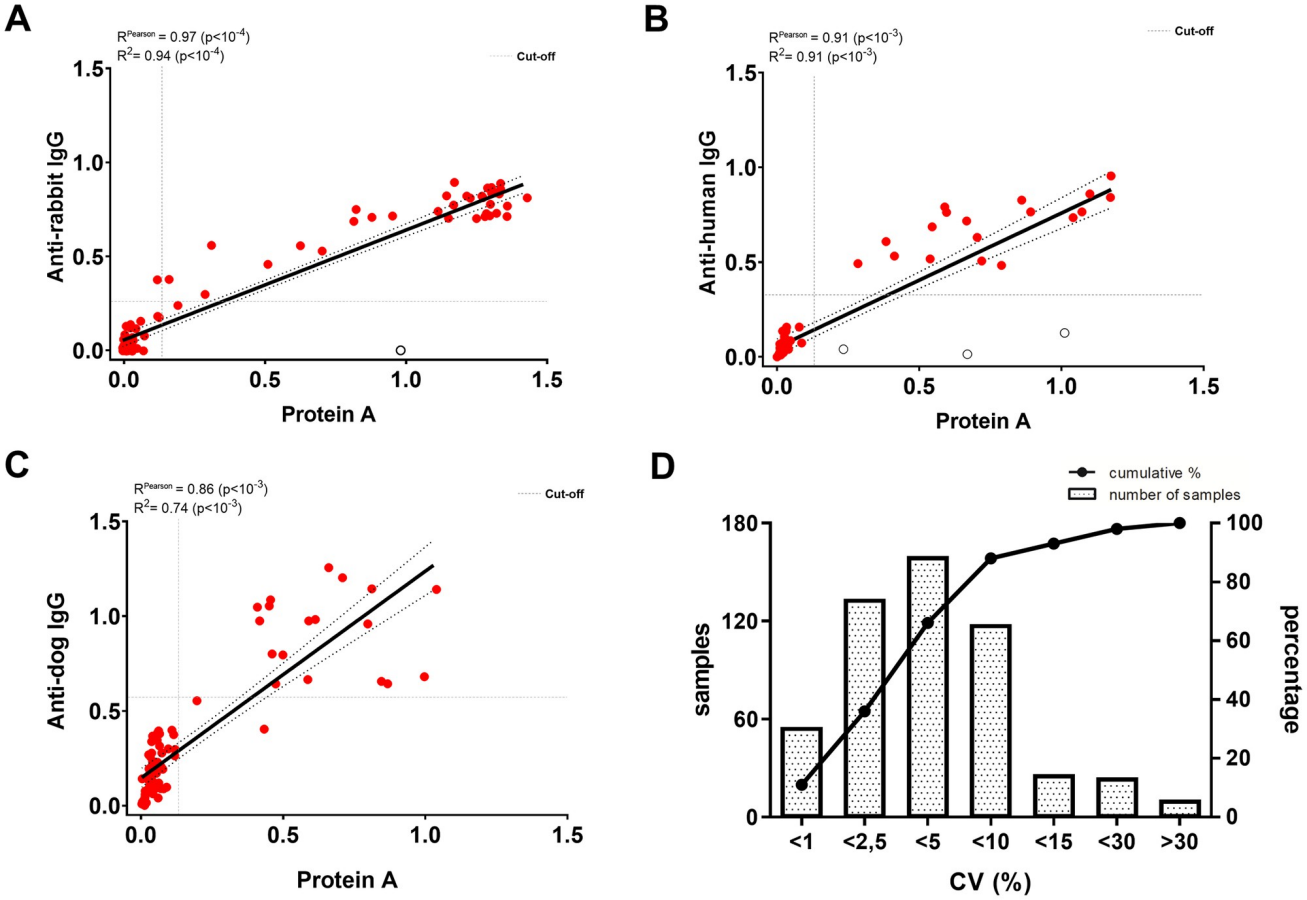
Linearity and precision of Protein A-ELISA. Correlation between the Protein A and IgG ELISA tests using control rabbit sera (n = 81) **(A)**; human sera (n = 64) **(B)** and dog sera (n = 77) **(C)**. Dashed lines represent the 95% confidence interval and the white data points represent outliers excluded from correlation analysis. From the total 553 samples tested for Protein A-ELISA, 88% had a coefficient of variation (CV) of the triplicates lower than 10% and 93% of the samples had a CV lower than 15%. The line shows the cumulative percentage of samples within the respective CV level and the bars show the absolute amounts of samples in each interval of CV value **(D)**.

We observed a good degree of correlation between ODs from Protein A and Anti-IgG ELISA methods (Fig 3A–3C). The mean ODs across six assay runs in different days were 1,392 (±0,069) for the positive rabbit serum (A1122) and 0,046 (±0,005) for the negative control, with a coefficient of variation (CV) of 4.9% and 10.8%, respectively. The repeatability, determined by eight intra-assay replicates, showed a CV of 0.5% for the positive anti-*Y*. *pestis* A1122 serum and 4.6% for the negative serum. Moreover, the intra-assay analysis of the triplicates from all samples tested for Protein A-ELISA revealed that 93% of the samples had a CV lower than 15% (Fig 3D). Of note, samples with CV > 15% were mostly negative sera with ODs close to the basal absorbance (blank, OD = 0.040–0.060), in a range where the background variations of the optical readings results in mathematical distortions in the CV formula (S2 Fig).

To evaluate whether these ELISA methods would present cross-reaction with other pathogenic yersiniae, sera from seven rabbits previously immunized with formol-killed *Y*. *pseudotuberculosis* (five) or *Y*. *enterocolitica* (two) strains were tested. Although the average ODs from these sera were slightly higher than other negative samples (protein A: 0.076 *versus* 0.014 and IgG: 0.133 *versus* 0.031), only one from the seven tested samples (*Y*. *enterocolitica*) presented a false-positive result for Protein A-ELISA (Fig 2A and 2B).

### Performance of Protein A-ELISA and Anti-IgG ELISA

Taking into consideration the HA results for 98 positive and 190 negative reference samples, the analysis of protein A and IgG ELISAs performance rates revealed high sensitivity, specificity and accuracy rates for both methods (Table 1). The Protein A-ELISA test had two false-positives (one rabbit and one dog) and six false negatives (four rodents, one rabbit and one dog), with an overall sensitivity of 93.9% and specificity of 98.9%. On the other hand, the Anti-IgG ELISA test had six false-positives (three humans, two dogs and one rabbit), with a sensitivity of 97.4% and specificity of 100% for rabbits, 93% and 100% for humans and finally, 90% and 100% for dogs. Confirming these findings, the ROC curves from both protein A and IgG methods showed high area under the curve (AUC) rates. Whereas the overall and species-specific AUCs from protein A-ELISA tests remained above 0.990, AUCs from Anti-IgG ELISA ranged from 0.930 to 0.982 (Fig 2C and 2D, and S3 Fig).

The Kappa test was initially applied to measure the degree of reliability between the ELISA tests and the HA (n = 288 for protein A and n = 222 for IgG). Excellent agreement rates were observed in samples from all species (Table 1). Next, we included 265 independent samples with unknown HA results (and not used in cut-offs calculation) and calculated the kappa index to measure the agreement between Protein A and IgG ELISAs (Table 2). From the 487 samples, 84 were positive in both tests, 398 were negative in both tests, eight were positive for protein A but negative for IgG and five were positive for IgG but negative for protein A. Kappa coefficient for all species was 0.905 (0.854–0.956), for rabbits: 0.925 (0.842–1.000), for humans: 0.914 (0.818–1.000) and for dogs: 0.850 (0.741–0.959). The p-values were < 0.05 in all tests.

**Table 2:** Kappa agreement test between Protein A-ELISA and Anti-IgG ELISA.

Species	n	Observed agreements (%) *	Kappa (ProtA vs IgG)
Rabbits	81	78 (96.3)	0.925 (0.842–1.000)
Humans	186	183 (98.4)	0.914 (0.818–1.000)
Dogs	220	213 (96.8)	0.850 (0.741–0.959)
All	487	474 (97.3)	0.905 (0.854–0.956)

* The cut-offs used to perform this test were the same established with the HA-tested samples, available at Table 1.

* Rodents were not included.

**Fig 4:**
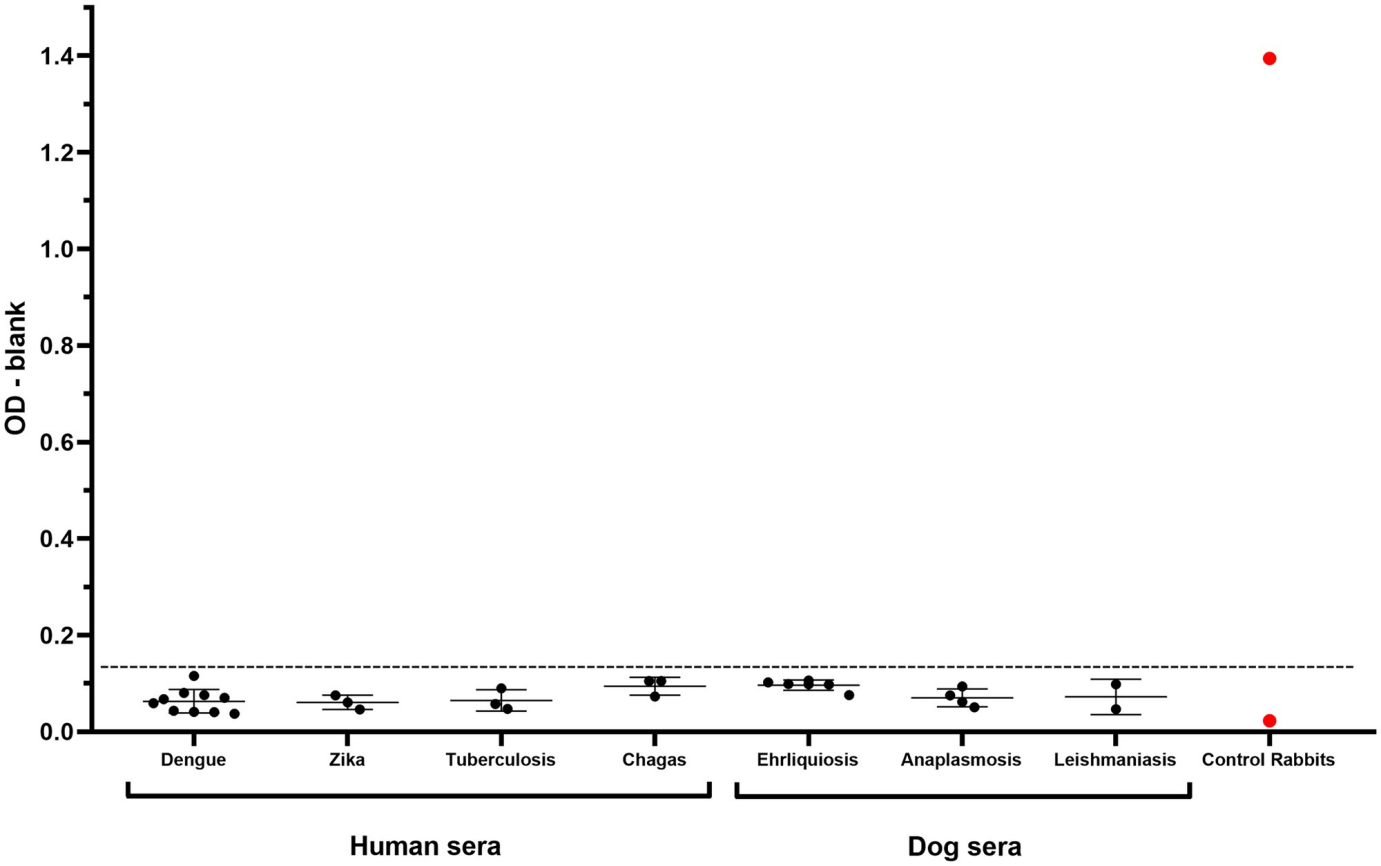
Evaluation of cross-reactivity of the ELISA Protein A method with other common infectious diseases. OD from sera from individuals positive for other infectious diseases, such as dengue (n = 10), Zika (n = 3), tuberculosis (n = 3) and Chagas disease (n = 3) (humans) and ehrlichiosis (n = 6), anaplasmosis (n = 4) and leishmaniasis (n = 2) (dogs). The red data points indicate the positive and negative controls (rabbit sera) and the error bars represent the average values with the 95% confidence interval.

Additionally, we investigated whether the Protein A-ELISA assay for plague would produce cross-reactivity with other endemic pathogens, by testing human and dog samples with confirmed infections and observed no cross-reaction with these conditions (Fig 4).

## Discussion

The gold standard for plague diagnosis is the identification and isolation of the *Y*. *pestis* in bacteriological cultures from the clinical specimen. However, as proper diagnosis is often not feasible due to the acute progression of the disease and geographic isolation of cases, patients frequently receive treatment without laboratory results [2,12]. Therefore, serological diagnosis is of most importance for plague diagnosis and surveillance activities as it can retrospectively identify humans and other hosts exposed to the bacteria [9,10]. In this scenario, serological surveillance must consider a wide variety of mammals to be tested, such as rodents and other small mammals, domestic (dogs and cats) and wild carnivores that prey on rodents [5,12,13,22,23].

Here, we describe a Protein A-based approach designed to overcome some limitations faced by routine laboratories when using other serological methods, such as HA (subjective interpretation, high consumption of antigen, perishable reagents) and conventional ELISA (requires specific IgG-peroxidase conjugate, cut-off calculation and positive controls for each species). While the protocol here established requires 750 ng of F1 antigen per tested sample (using triplicates), HA-HI spends up to 20,000 ng of F1 per tested sample (considering the standard eight dilutions according to Chu) [10], resulting in the use of approximately twenty-seven times more antigen per sample. This difference can be particularly relevant for plague diagnosis given the complexity and costs of producing and purifying F1 from extensive *Y*. *pestis* culturing in biosafety level 3 (BSL3) laboratories [27].

Throughout a broad range of host species hereby tested, the Protein A-ELISA method showed high sensitivity, specificity and reproducibility rates using a single cut-off value for all species. The analysis of the ROC curve showed AUCs above 0.990 in all groups tested in protein A-ELISA, while AUCs from Anti-IgG ELISA ranged from 0.930 to 0.982. The Cohen’s Kappa test revealed high agreement rates for this protocol when compared to HA (n = 288) and Anti-IgG ELISA (n = 487). Because human cases of plague have not been reported in Brazil since 2005, we were not able to estimate positive/negative predictive values [31]. It is important to highlight that, since performance rates were calculated according to the results obtained by HA, eventual unnoticed cases of false positives/negatives in the HA-referenced sample set may bias the determination of sensitivity, specificity and AUCs, depreciating the outcome of these parameters for the ELISA method.

Remarkably, we observed a good correlation between ODs from Protein A and anti-IgG, with higher positive/negative OD ratios in the Protein A-ELISA test, which allows a safer window of opportunity for cut-off determination between positive and negative samples. Of note, little cross-reaction was observed in sera from rabbits immunized with other pathogenic yersiniae. Additionally, we observed no cross-reaction in the Protein A test with human and dog sera from individuals positive for other common infectious diseases. Interestingly, whilst negative samples showed low background signals in Protein A, anti-rabbit and anti-humans IgG conjugates, a rather marked background in anti-dog IgG conjugate was observed. This could be associated with the non-specific agglutination routinely observed in sera from dogs in diagnosis by HA.

Although remaining detectable in humans for several years after infection, antibodies against *Y*. *pestis* usually can be detected from the fifth day of infection by HA and from the eighth day by Anti-IgG ELISA [16,32,33]. Of interest, the two human cases with the shortest interval between symptoms onset and serum collection (2 and 6 days) in our study showed a specific pattern: both tested positive in HA and Protein A-ELISA, but negative in Anti-IgG ELISA. This finding suggests that, similarly to HA, the Protein A system is capable of recognizing IgM during acute phase, before serum conversion to IgG. Although this result is preliminary and must be interpreted with caution, it is supported by previous studies showing that in addition to its affinity for IgG from a wide range of mammals, protein A can also bind to IgA, IgM and IgE [34,35].

The Protein A-ELISA had good sensitivity/specificity for the rodents’ group, however, we were able to access positive samples from only two species: *M*. *musculus* (Muridae) and *C*. *callosus* (Cricetidae). This underrepresentation of the diversity within the Rodentia order is an important limitation of this study, as the Protein A has variable affinity to antibodies across the rodent’s species. This particularity can potentially impact the sensitivity of the test for species with low protein A binding capacity, such as *R*. *rattus* [36]. Therefore, further studies are necessary to evaluate this approach in a broader selection of not only rodents, but also other wild mammal species.

Altogether, we evaluated a Protein A-based indirect ELISA test that is sensitive, specific and reproducible, with a single protocol that can be used for both diagnosis of plague in humans and epidemiological surveillance in animal reservoirs and sentinels from active foci.

## Supporting information

S1 Fig Standardization of Anti-IgG ELISA. Four distinct titers were tested for each anti-IgG conjugates in triplicate for one positive and one negative serum from each species. Antigen concentration and sample titer were the same from the previously established in the Protein A-ELISA assay.
(TIF)

S2 Fig Coefficient of variation in Protein A-ELISA according to the average OD. The majority of the samples tested for Protein A-ELISA (n = 553) had triplicates with low CVs. Higher variation was found in negative samples, with the ODs close to the lower detection limit, where small numeric variations imply in high CVs.
(TIF)

S3 Fig ROC curves for Protein A-ELISA. Stratified ROC curves and area under the curve (AUC) for protein A-ELISA according to each evaluated species.
(TIF)

S4 Fig Hemagglutination titers from the human positive sera. The right vertical axis shows the distribution of the 21 positive human sera according to their HA titers (bars in gray) and the left vertical axis indicate the OD values obtained in the ELISA Protein A for each group of samples with a specific HA titer (data points). The error bars show the range of the ODs for each group.
(TIF)

S1 Table Average ODs for positive and negative samples for Protein A and IgG ELISA. (DOCX)

S2 Table Epidemiological and laboratorial data from the 21 human cases of plague evaluated in the study. (XLSX)
