# Supplementary figures and images for: Evaluation of a multi-species Protein A-ELISA assay for plague serologic diagnosis in humans and other mammal hosts
Source: PLoS Negl Trop Dis. 2022 May 12;16(5):e0009805. doi: 10.1371/journal.pntd.0009805 (PMC9129028; doi:10.1371/journal.pntd.0009805)

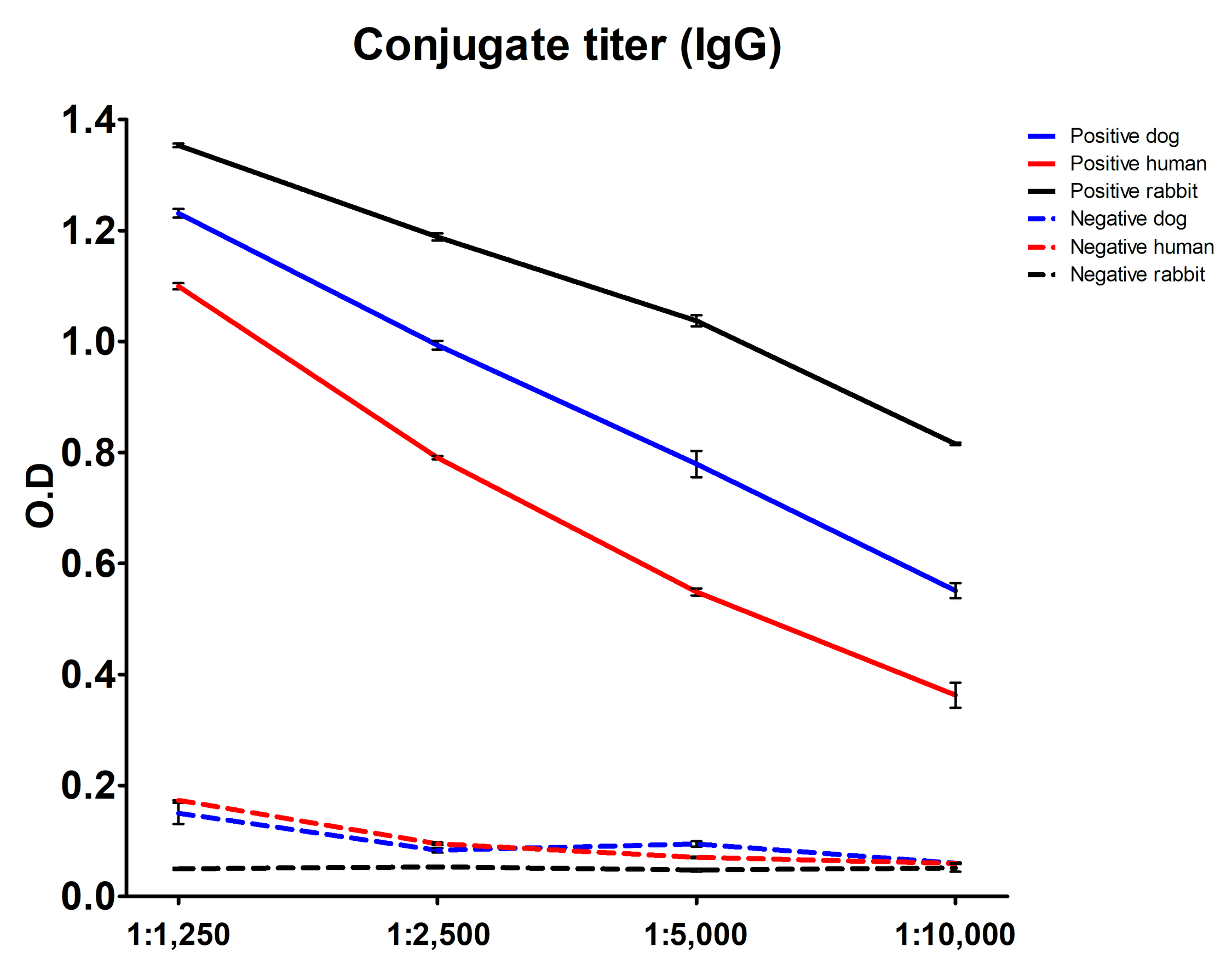

Supplement: S1 Fig — Four distinct titers were tested for each anti-IgG conjugates in triplicate for one positive and one negative serum from each species. Antigen concentration and sample titer were the same from the previously established in the Protein A-ELISA assay. (TIF) [file pntd.0009805.s001.tif]

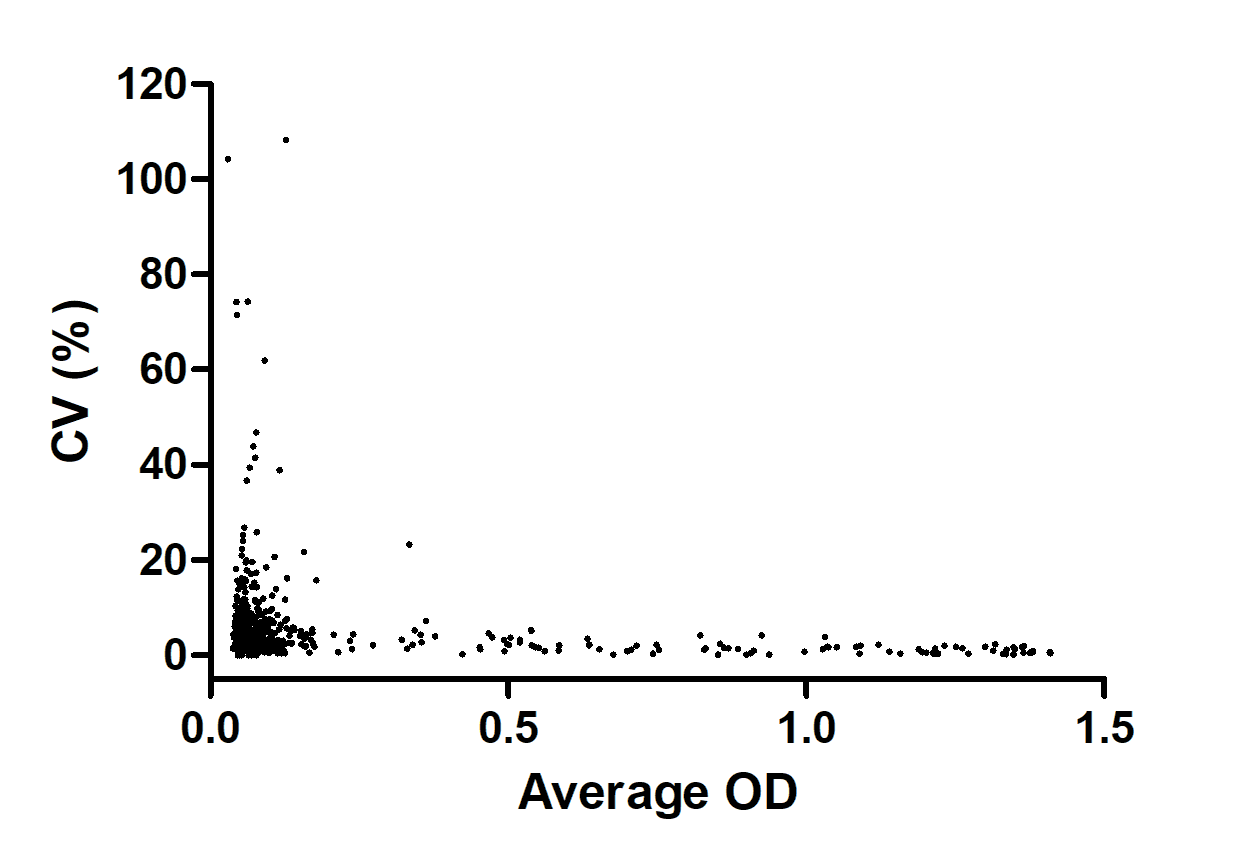

Supplement: S2 Fig — The majority of the samples tested for Protein A-ELISA (n = 553) had triplicates with low CVs. Higher variation was found in negative samples, with the ODs close to the lower detection limit, where small numeric variations imply in high CVs. (TIF) [file pntd.0009805.s002.tif]

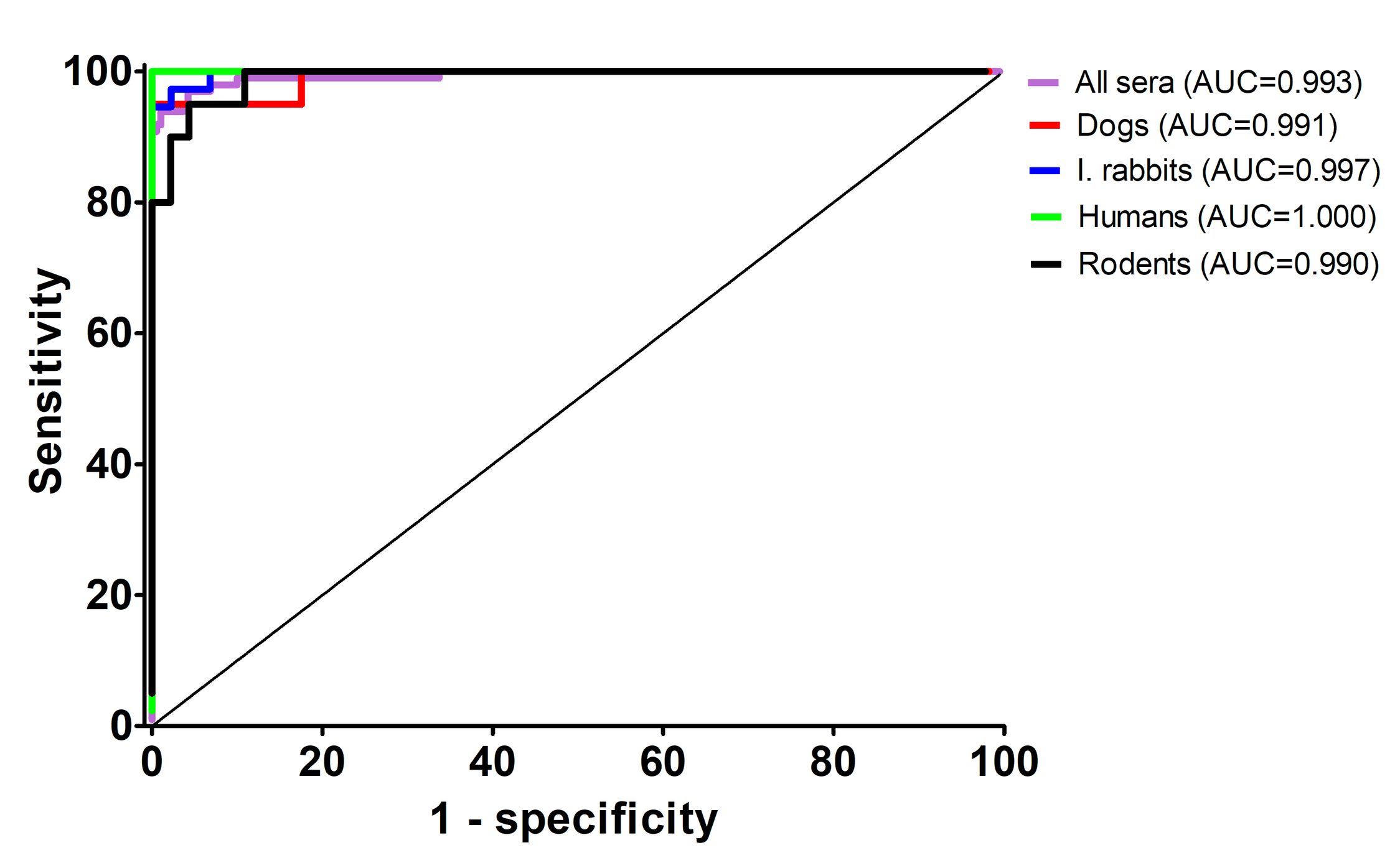

Supplement: S3 Fig — Stratified ROC curves and area under the curve (AUC) for protein A-ELISA according to each evaluated species. (TIF) [file pntd.0009805.s003.tif]

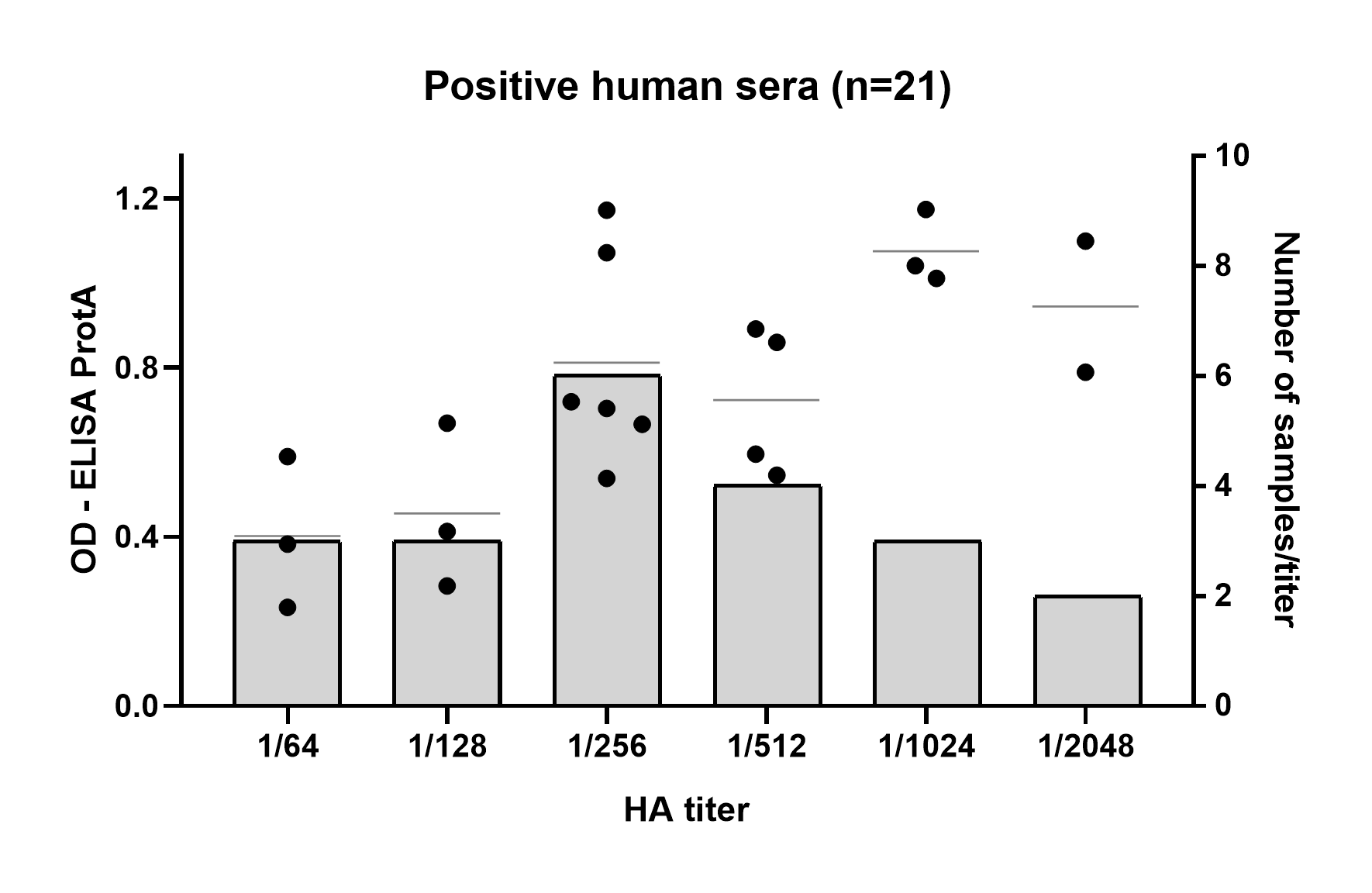

Supplement: S4 Fig — The right vertical axis shows the distribution of the 21 positive human sera according to their HA titers (bars in gray) and the left vertical axis indicate the OD values obtained in the ELISA Protein A for each group of samples with a specific HA titer (data points). The error bars show the range of the ODs for each group. (TIF) [file pntd.0009805.s004.tif]
