# Supplementary material for: Evaluation of a multi-species Protein A-ELISA assay for plague serologic diagnosis in humans and other mammal hosts
Source: PLoS Negl Trop Dis. 2022 May 12;16(5):e0009805. doi: 10.1371/journal.pntd.0009805 (PMC9129028; doi:10.1371/journal.pntd.0009805)
Supplement: S1 Table — (DOCX) [file pntd.0009805.s005.docx]

**Supplementary Table 1**. Average ODs for positive and negative samples for Protein A and IgG ELISA.

|  | Positive (CI95%)* | Negative (CI95%) |
| --- | --- | --- |
| Protein A |  |  |
| Rabbits | 1.054 (0.903-1.177) | 0.0138 (0.007-0.020) |
| Rabbits (*Y.enterocolítica/Y.tuberculosis*) | - | 0.0760 (0.013-0.140) |
| Human | 0.735 (0.604-0.866) | 0.022 (0.016-0.027) |
| Rodent | 0.466 (0.305-0.628) | 0.009 (0.001-0.017) |
| Dog | 0.609 (0,499-0,719) | 0.048 (0.038-0.057) |
| All | 0.775 (0.695-0.854) | 0.027 (0.022-0.032) |
| Anti-IgG |  |  |
| Rabbits | 0.695 (0.630-0.760) | 0.031 (0.017-0.045) |
| Rabbits (*Y.enterocolítica* and *Y.tuberculosis*) | - | 0.133 (0.0690.197) |
| Human | 0.602 (0.482-0.722) | 0.051 (0.039-0.063) |
| Dog | 0.865 (0.736-0.995) | 0.178 (0.145-0.212) |

^*Samples were classified as positive or negative according to results from HA (n=288).^
